# Neutrophil extracellular traps are downregulated by glucocorticosteroids in lungs in an equine model of asthma

**DOI:** 10.1186/s12931-017-0689-4

**Published:** 2017-12-12

**Authors:** Amandine Vargas, Roxane Boivin, Patricia Cano, Yoana Murcia, Isabelle Bazin, Jean-Pierre Lavoie

**Affiliations:** Department of Clinical Sciences, Faculty of Veterinary Medicine, Université de Montréal, 3200, rue Sicotte, Saint-Hyacinthe, Quebec J2S 2M2 Canada

**Keywords:** Neutrophil activation, Neutrophil extracellular traps and glucocorticosteroids

## Abstract

**Background:**

Severe neutrophilic asthma is poorly responsive to glucocorticosteroids (GC). Neutrophil extracellular traps (NETs) within the lungs have been associated with the severity of airway obstruction and inflammation in asthma, and were found to be unaffected by GC in vitro. As IL-17 is overexpressed in neutrophilic asthma and contributes to steroid insensitivity in different cell types, we hypothesized that NETs formation in asthmatic airways would be resistant to GC through an IL-17 mediated pathway.

**Methods:**

Six neutrophilic severe asthmatic horses and six healthy controls were studied while being treated with dexamethasone. Lung function, bronchoalveolar lavage fluid (BALF) cytology and NETs formation, as well as the expression of CD11b and CD13 by blood and airway neutrophils were evaluated. The expression of IL-17 and its role in NETs formation were also studied.

**Results:**

Airway neutrophils from asthmatic horses, as opposed to blood neutrophils, enhanced NETs formation, which was then decreased by GC. GC also tended to decrease the expression of CD11b in blood neutrophils, but not in airway neutrophils. IL-17 mRNA was increased in BALF cells of asthmatic horses and was unaffected by GC. However, both GC and IL-17 inhibited NETs formation in vitro.

**Conclusion:**

GC decreased NETs formation in vitro and also in vivo in the lungs of asthmatic horses. However, airway neutrophil activation during asthmatic inflammation was otherwise relatively insensitive to GC. The contribution of IL-17 to these responses requires further study.

**Electronic supplementary material:**

The online version of this article (10.1186/s12931-017-0689-4) contains supplementary material, which is available to authorized users.

## Background

Asthma is characterized by airflow obstruction primarily due to bronchospasms, chronic inflammation and remodeling of the airway wall. Glucocorticosteroids (GC) are the most effective drugs used to control asthma symptoms and exacerbation. However, neutrophils the predominant cells within the airways of a subset of asthmatic patients, are recognized as poor responders to GC treatment even with high doses of inhaled glucocorticosteroids (ICS). Indeed, GC significantly decrease the recruitment and activation of several inflammatory cells (mast cells, eosinophils, macrophages and T lymphocytes) in asthmatic airways, yet pulmonary neutrophilia remains unchanged [1–3]. However, blood neutrophils are sensitive to the effects of glucocorticoids in vitro, suggesting that the activation stimuli occurring within in airways are responsible for the lack of inhibition of airway neutrophilia observed in asthma under GC treatment [4].

Recently, NETs have emerged as a new microbial and cytotoxic mechanism of the immune response to infections and injuries [5, 6]. NETs are formed by decondensed chromatin and bactericidal proteins released by granules of neutrophils, through a process called NETosis [7]. This release is induced by several agents (reactive oxygen species, bacteria, fungi, viruses, antigen–antibody complexes, microbial components and lipopolysaccharide) [8–13] and leads to tissue injury [5, 6]. Several other factors, such as *N*-formyl-methionyl-leucyl-phenylalanine (fMLP) can induce neutrophil activation without leading to NETs formation [14]. Indeed, unlike previous beliefs, neutrophils have been shown to modulate their responses according to the mediators present in their environment [15], and therefore the contribution of disease processes is complex. NETs are associated with inflammation and disease severity in chronic airway diseases such as asthma [16], and cause airway obstruction [17]. In sputum of patients with chronic obstructive pulmonary disease **(**COPD), however, NETs formation is present whether the patients are in exacerbation or not [18]. While GC (dexamethasone) did not prevent NETs formation induced by stimulating peripheral blood neutrophils with phorbol myristate acetate (PMA) in vitro [19], no study, to the best of our knowledge, has investigated NETs formation in vivo during treatment using these drugs.

Despite a recent interest in the role of NETs in disease pathogenesis, the molecular mechanisms resulting in NETs formation and their regulation remain unknown [20]. It was recently shown in an immune-mediated disorder, that neutrophils release IL-17 through NETs formation [21]. IL-17 is considered to play a central role in severe asthma and to contribute to neutrophilic inflammation and steroid insensitivity [22], particularly in asthmatics with frequent exacerbations [23]. We previously found that this cytokine induces the activation of equine neutrophils and importantly, that it is not inhibited by GC [24]. However, IL-17’s contribution to NETs formation and its insensitivity to GC have not been studied in asthma. We therefore hypothesized that NETs formation in asthmatic airways would be resistant to GC through an IL-17 mediated pathway. We also postulated that the IL-17 rich lung microenvironment in asthma would contribute to this insensitivity. We investigated NETs in both airway and blood neutrophils of asthmatic horses before and during GC administration. We studied horses with severe neutrophilic asthma (severe equine asthma, also known as heaves), a condition that shares marked similarities with human neutrophilic asthma [25], including the remodeling of the ASM layer [26] and extracellular matrix [27]. Furthermore, human and equine neutrophils have similar biology [4, 24, 28].

## Methods

### Study design

Twelve adult horses including eight mares and four castrated males were studied (Table 1). Six horses with severe asthma had a history of recurrent episodes of airway obstruction (pulmonary resistance (R_L_) > 1 cm H_2_O/L/s), and increased neutrophils (≥ 25%) in bronchoalveolar lavage fluid (BALF) upon antigen exposure. Age-matched control horses (*n* = 6) had no history of respiratory diseases, normal lung function (R_L_ < 1 cm H2O/L/s) and <15% of neutrophils in BALF with stabling and hay feeding. Horses were deemed otherwise healthy. All horses were antigen exposed by being stabled in the same barn and fed hay for 1 month, as these conditions are known triggers of disease exacerbation in susceptible animals [25]. Horses were then treated with dexamethasone (Dominion Veterinary Laboratories ltd, MB, CA) at a dosage of 0.06 mg/kg orally, once daily for 2 weeks. Blood and bronchoalveolar lavages were collected at the Baseline and after one and two weeks of treatment. All experimental procedures were performed in accordance with the Canadian Council for Animal Care guidelines and were approved by the Animal Care Committee for the Faculty of Veterinary Medicine of the Université de Montréal (deontology Rech-1716).

**Table 1:** Summary table of animal characteristics. F, females; M, castrated males

Sex	Age (years)	Medical status	Weight (kg)
F	23	Asthma	448
M	18	Asthma	437
M	12	Asthma	560
M	10	Asthma	586
M	25	Asthma	522
M	7	Asthma	507
F	13	Control	467
F	13	Control	525
F	18	Control	447
F	10	Control	534
F	18	Control	452
M	5	Control	440

### Bronchoalveolar lavages fluid (BALF)

BALF collection was performed as previously described [29]. For NETs quantification (score), BALF was centrifuge at 1000 rpm and resuspend in 50 ul PBS. Thin-layer cells from BALF (or 50 ul for NETs score) were prepared with a cytocentrifuge (Rotorfix, Hettich, Berlin, DEU) and stained with a modified Wright–Giemsa solution (DiffQuick, Fisher Scientific, Waltham, Massachusetts, USA) for cytology or fixed in paraformaldehyde 4% for immunofluorescence evaluation. Differential cell counts were obtained from 400 cells. The remaining BALF were then centrifuged at 1600 rpm at 4 °C for 5 min and cells were resuspended in PBS 1X for flow cytometry analysis and in Isol-RNA solution (Fisher Scientific, Ottawa, ON, CA) for gene expression analysis.

#### NETs quantification

**Fig. 1:**
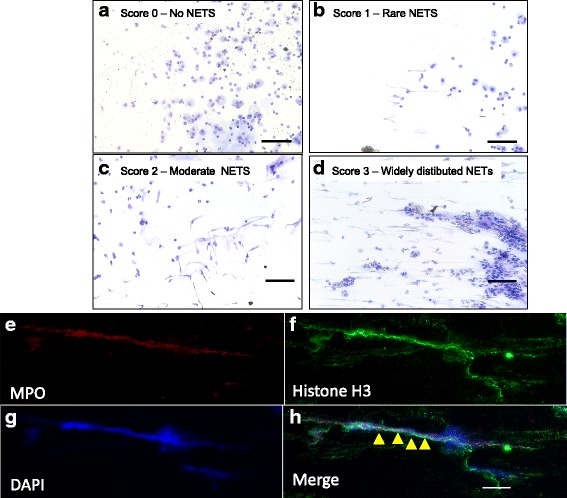
Scoring system for BALF NETs quantification. **a**-**d** NETs on cytospins BALF were evaluated using a blind scoring system. Scores 0 (no NETs), 1 (rare NETs), 2 (moderate NETs) and 3 (widely distributed NETs) were attributed to each of the 27 fields from the peripheral areas of the cytospin. Scale bars represent 100 μm. **e**-**h** Representative images illustrating NETs in the BALF of a horse with severe asthma. BALF cells were labeled with anti-myeloperoxidase antibody (red), anti-Histone H3 (Green) and DAPI (nucleus; blue), then observed by fluorescence microscopy at a magnification of 200X. Scale bar represents 10 μm. Arrowheads indicate profuse NETs appearing as a web*-*like extracellular structure

NETs were evaluated on cytospins of BALF stained with Wright-Giemsa using a Leica microscope (DM4000B Leica Microsystems, Wetzlar GmbH, DEU) under polarized light with a 200X magnification. Each slide was divided into 27 equal fields using the Panoptiq software (ViewsIQ, Richmond, BC, CA, v1.4.3). A blind quantification of NETs was performed by attributing a score of 0 (no NET), 1 (rare NETs), 2 (moderate NETs) and 3 (widely distributed NETs) to all fields of the peripheral areas of the cytospins, as this is where NETs are concentrated (Fig. 1a-d). The final value per horse was obtained by calculating the mean NETs score of its slide, normalized by neutrophil count in the same fields. Two BALF cytospin slides for each horse and at each time point of the study were counted. Score was validated by comparing the staining of the extracellular DNA regions using Sytox Orange, then using an unbiased point counting approach (2304 points per 20X field, with each point corresponds to a surface of 0.37595 mm^2^) as described in the Additional file 1: Figure S1 and Additional file 2, on BALF cytospin with an immunofluorescence staining (please see below the immunofluorescence section).

#### Apoptosis

Percentage of apoptotic neutrophils was determined on 500 neutrophils randomly distributed in each cytospin slide under a light microscope at 400X magnification. When total count of neutrophils was below 500, all neutrophils in the slide were included in the analysis. Apoptotic neutrophils were defined by the presence of one or more pyknotic nuclear remnants, previously shown to be well correlated with specific apoptotic marker (i.e. Annexin V staining) (3).

#### Immunofluorescence

NETS on BALF cytospins were also evaluated using immunofluorescence. Cytospins fixed in paraformaldehyde were washed with PBS and incubated in the presence of rabbit polyclonal anti-myeloperoxidase antibody (MPO) (Dako; Mississauga, ON, CA, dilution 1/200) and mouse monoclonal anti-Histone H3 (Abcam; Toronto, ON, CA 1/200) in PBS containing 0.2% bovine serum albumin and 0.1% Triton X-100 (Sigma-Aldrich, Oakville, ON, CA) for 1 h 30 at room temperature, then with the Alexa Fluor 488-conjugated goat anti-rabbit IgG (Thermofisher Scientific, Burlington, ON, CA; dilution 1/1000) for 1 h at room temperature. For nuclear staining, cells were incubated with DAPI (Thermofisher Scientific, Burlington, ON, CA; 300 nM) for an additional 5 min at room temperature. After three washes with PBS, the coverslips were then mounted in a drop of ProLong Antifade reagent (Thermofisher Scientific, Burlington, ON, CA) and visualized with a Zeiss (Axio Imager M1) fluorescence microscope. All observations were performed at a final magnification of 200X. A NET was defined as a network-like extracellular structure (Fig. 1e-h).

#### Flow cytometry

Total BALF cells were studied by flow cytometry using neutrophil activation surface markers anti-CD11b and anti-CD13. Cells (10^6^cells/100 μl) were stained for 1 h with anti-CD11b (Mylteni Biotec Auburn, USA; dilution 1/10) or anti-CD13 (Ab Serotec respectively, Raleigh, USA dilution 1/10). Cells were then incubated 10 min with PBS containing 0.1% Triton X-100 (Sigma-Aldrich, Oakville, ON, CA) and stained for 1 h with and anti-MPO (Dako; Mississauga, ON, CA, dilution 1/200). All incubation steps were performed at 4 °C. Cells were then washed 3 times in washing buffer and incubated 30 min in the dark with Alexa488- or Alexa594-conjugated anti-IgG antibodies (Thermofisher Scientific, Burlington, ON, CA; dilution 1/1000) depending on the first staining (CD11b-FITC or CD13-PE). Cells were washed twice and resuspended in 400 μl PBS before flow cytometry acquisition of 10,000 events and analysis using BD Accuri C6 software and instrument (BD Bioscience, San Jose, CA, USA). Isotype-matched control antibodies (Polyclonal Rabbit IgG, rat IgG2a-PE and mouse IgG-FITC; Vector Laboratories, Burlington, ON, CA, Mylteni Biotec Auburn, USA and Ab Serotec, Raleigh, USA, respectively) were used as controls. Staining was evaluated by the mean percentage of positive cells.

#### Real-time RT-PCR (qRT-PCR)

The expression of IL-17A was measured in BALF cells by qRT-PCR for each time point of the study. mRNA was extracted using Isol-RNA Lysis Reagent according to manufacturer’s instructions (Fisher Scientific, Ottawa, ON, CA). Purity and concentration were assessed using a spectrophotometer Nanodrop ND1000 (Fisher Scientific, Waltham, MA, USA). Total RNA (1 μg) was reverse transcribed as described previously [4]. The qRT-PCR was performed using specific primers (Table 2), and PCR products were sequenced to ensure the specificity of amplifications. Quantitative PCR (qPCR) was performed by monitoring the increase of fluorescence of SYBR® Green in real-time (Bio-Rad, Hercules, CA, USA) with the CFX96 Touch Real-Time PCR Detection System (Bio-Rad, Hercules, CA, USA). The gene expression was normalized with the CFX Manager™ Software (Bio-Rad, Hercules, CA, USA) using RPL9 (Ribosomal Protein L9) as reference gene. Samples were run in duplicate with an appropriate negative control.

**Table 2:** Sequences of primer pairs used for quantitative PCR analysis

5′- AATCCGGAATGCCGAACAC −3′	IL-17A forward
5′- CTACCTTCCCTTCGGCATTG −3′	IL-17A reverse
5′- TCTCAGCAATCAGACCGTG −3′	RPL9 forward
5′- TGTCAACTCGGAGCCTCTTC −3′	RPL9 reverse

### Blood neutrophils

#### Blood neutrophils isolation

Neutrophils were isolated from EDTA anti-coagulated blood through centrifugation on a density gradient using Ficoll-Paque (Ficoll-PaqueTM PREMIUM 1084, GE Healthcare Bio-sciences Corp, Mississauga, ON, CA) according to the manufacturer’s instructions. The remaining red blood cells were lysed with distilled water. Cell counting and viability was performed using the ADAM automatic Cell Counter (Montreal-Biotech Inc., Montreal, QC, CA). Cytospin slides were prepared and stained with a modified Wright–Giemsa solution (DiffQuick) for differential counting on 400 cells to assess neutrophil purity. The purity and viability of neutrophils were >99% and >95%, respectively. Neutrophils were suspended at 5 × 10^6^ cells/ml in culture medium RPMI 1640 supplemented with 10% heat inactivated low-endotoxin FBS, 2 mM l-glutamine, 100 U/mL penicillin, and 100 μg/mL streptomycin (all products from GIBCO, Thermofisher Scientific, Burlington, ON, CA) for in vitro experiments or fixed in paraformaldehyde 4% for flow cytometry.

#### Evaluation of in vitro NETs formation

In one experiment, isolated blood neutrophils were seeded (1 × 10^6^) onto 24-well plates (non-treated plastic, Ultident, St-Laurent, QC, CA) either in the presence or absence of 100 ng/ml reIL-17 (Cederlane, Burlington, ON, CA) for 24 h. Neutrophils were stained with a modified Wright–Giemsa solution, and analyzed using the previously validated NETs score, and normalized for cell density using an automatic cell counting plugin in ImageJ [30].

In other experiment, real-time NETs neo-formation was evaluated by incubating neutrophils either in the presence or absence of PMA (200 nM, Sigma-Aldrich, Oakville, ON, CA), +/−100 ng/ml reIL-17 (Cederlane, Burlington, ON, CA) and 10^−6^ M dexamethasone (Sigma-Aldrich, St Louis, MO, USA) for 1 h. Cells were stained with the cell-impermeable fluorescent DNA-staining Sytox (2.5 μM, Thermofisher Scientific, Burlington, ON, CA) and immunostained (see immunofluorescence section below). Ten images were randomly taken at 100X magnification using a Leica microscope (DMIRB model) by a blinded evaluator. This magnification with a non-invert microscope was more appropriate for in vitro experiments and allows a better brightness and color discrimination with the 24-well plastic plates. Images were also subsequently blindly analyzed with the NETs score and results were then expressed as fold change relative to PMA stimulated cells.

#### Immunofluorescence

Stimulated neutrophils were seeded (1 × 10^6^) onto six-well plates containing 1.5 mm-thick poly-L-lysine-coated coverslips. Cells were fixed in paraformaldehyde 4% for 20 min and incubated in PBS containing 2% FBS (*v*/v) for 30 min to eliminate non-specific binding, then immunofluorescence for MPO, histone H3 and DAPI was done as described above.

#### Flow cytometry

Flow cytometry was performed on paraformaldehyde fixed blood neutrophils using neutrophil activation surface markers anti-CD11b and anti-CD13 as described above.

### Statistical analysis

Data were statistically evaluated using a linear model that includes repeated measures using time as a within-subjects factor, and treatment as a between-subjects factor for all values. Post hoc analyses were performed using student *t*-tests with the alpha threshold of each comparison adjusted using the sequential Benjamini-Hochberg method (SAS software). The Spearman test was used to correlate scores and data generated with an unbiased point counting approach. The in vitro NETs formation was evaluated using a linear model with treatment as factor. A *p*-value of 0.05 was considered significant and all results were expressed as mean ± standard error of the means (SEM).

## Results

### Animals

**Fig. 2:**
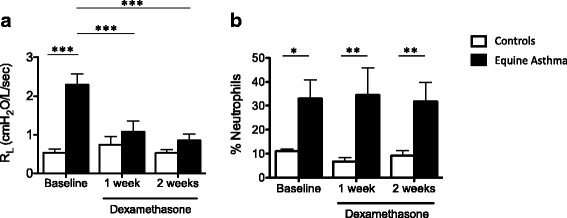
Pulmonary function and bronchoalveolar lavage fluid cytology. **a** Pulmonary resistance and (**b**) bronchoalveolar lavage fluid (BALF) differential neutrophil count of asthmatic (*n* = 6) and healthy (*n* = 6) horses before and during treatment with dexamethasone (0.06 mg/kg once daily). Mean ± SEM. *Different between groups. *p < 0.05, **p < 0.001 and ***p < 0.0001

Age and weight were not statistically different between groups (controls (mean ± SD): 12.8 ± 4.5 yrs. and asthmatic horses: 15.3 ± 6.6 yrs.; 510 ± 54 kg and 477 ± 38 kg; Table 1). Similarly, there were no differences between groups in terms of lung function and BALF cytology values when horses were in a low antigenic environment (pasture). Asthmatic horses under antigen exposure developed airway obstruction, whereas control horses had normal lung function (Baseline, Fig. 2a). Furthermore, the percentage of neutrophils in BALF was significantly elevated with antigen exposure in asthmatic horses when compared to control horses. After systemic dexamethasone administration, lung function markedly improved or normalized in asthmatic horses; however, the airway neutrophilia persisted (*p* = 0.003 after 1 week of treatment; *p* = 0.07 after 2 weeks of treatment; Fig. 2b).

### NETs score validation

The release of NETs was quantified using a validated visual scoring system. The score was significantly correlated with the unbiased point counting approach data (*r* = 0.79; *p* = 0.05, Additional file 1: Figure S1 (Additional file 2)).

### Airway neutrophils of asthmatic animals have enhanced NETs production

**Fig. 3:**
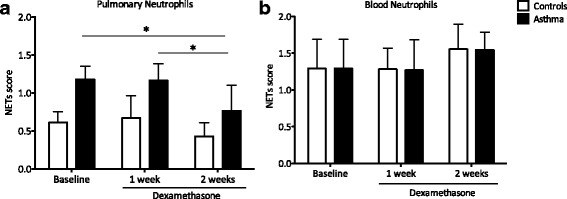
NETs evaluation during treatment with dexamethasone. NETs scores were evaluated in BALF (**a**) from control and asthmatic horses and in neutrophils isolated from blood (**b**) at all three time points (baseline, one week and two weeks). Results are presented as means of scores for each group ± SEM. Differences between groups *p < 0.05. The total numbers of neutrophils in blood and in the airway lumens remained unchanged

NETs scores at baseline were 2-fold higher in lungs of asthmatic horses compared to controls (*p* = 0.03), and dexamethasone decreased NETs formation only in asthmatic horses after two weeks of treatment (*p* = 0.02) (Fig. 3a). In blood, NETs scores were similar in both groups at all time points, and were not affected by GC (Fig. 3b).

### Pulmonary neutrophil activation is not affected by corticosteroid administration

**Fig. 4:**
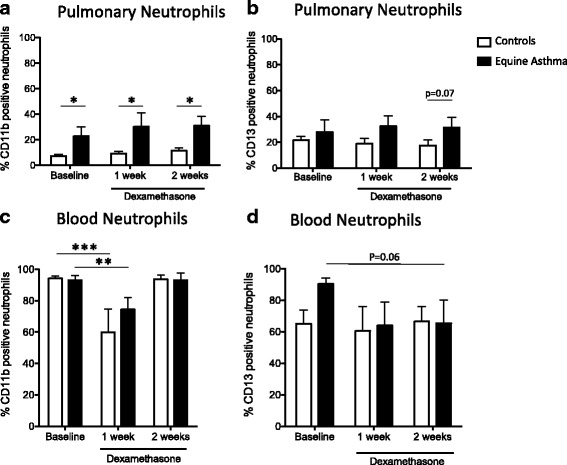
Pulmonary and blood neutrophil activation during treatment with dexamethasone. Pulmonary neutrophils (gating on the basis of size and granularity (FSC and SSC parameters) excluding macrophages, and MPO^+^ cells) were analyzed by flow cytometry for CD11b (**a**) and CD13 (**b**) at Baseline and after one and two weeks of treatment with GC. The percentages of CD11b^+^ and CD13^+^ of blood neutrophils were similarly evaluated for the two groups of horses (**c** and **d**). Each bar represents the mean ± SEM. Different between groups *p < 0.05, **p < 0.001 and ***p < 0.0001

We evaluated the expression levels of the two surface activation markers CD11b and CD13 on pulmonary and blood neutrophils (Fig. 4). The percentage of CD11b and CD13 positive neutrophils in the airways was 3 and 1.6-fold greater at Baseline in asthmatic horses when compared to healthy animals (Fig. 4a and b). The percentage of CD11b-positive neutrophils from asthmatic horses remained significantly higher compared to controls with therapy (*p* = 0.03 at Baseline; *p* = 0.05 after 1 week of treatment; *p* = 0.04 after 2 weeks of treatment; Fig. 4a). The expression of CD13 increased in asthmatic horses after two weeks of treatment; however, the difference did not reach significance (*p* = 0.07).

In blood, the expression level of CD11b decreased after one week of dexamethasone administration in both groups (Fig. 4c). While the percentage of CD13 positive neutrophils was not significantly different between groups (Fig. 4d), there was a trend towards a decrease after two weeks of GC treatment (*p* = 0.06).

### Corticosteroids decrease pulmonary neutrophil apoptosis

**Fig. 5:**
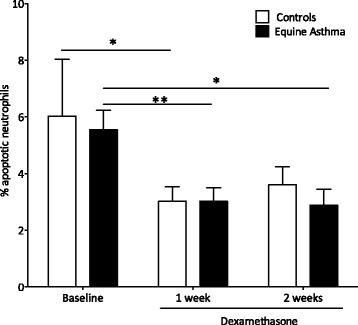
Pulmonary apoptotic neutrophils. Morphological features of pulmonary neutrophils apoptosis in BALF from control horses and from asthmatic horses were evaluated under a light microscope at 400X magnification. Bars represent means ± SEM. Different between groups *p < 0.05, **p < 0.001

We observed a significant decrease in pulmonary apoptotic neutrophils after one week of GC treatment in the control group (*p* = 0.04), and after one and two weeks in asthmatic horses (*p* = 0.01 and *p* = 0.005, Fig. 5).

### Corticosteroids have no effect on IL-17 expression in asthmatic animals

**Fig. 6:**
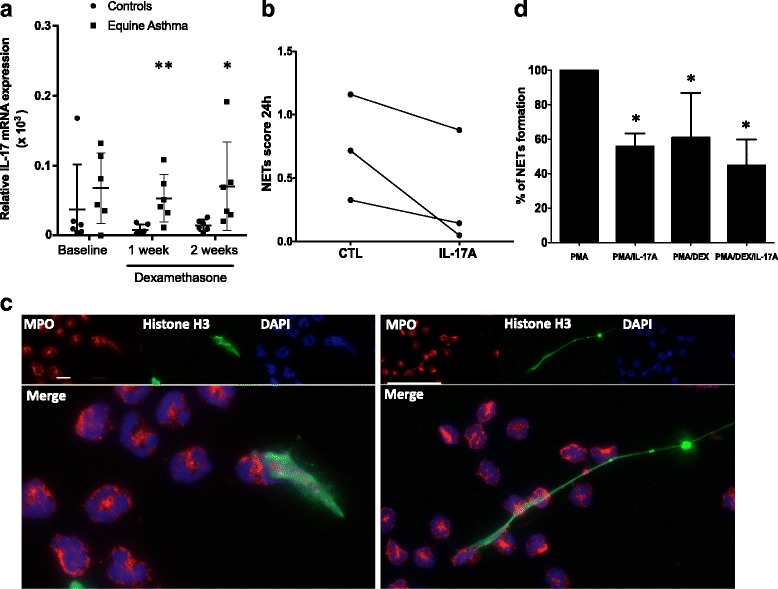
IL-17 effects on the PMA-induced release of NETs. **a** The mRNA expression of IL-17 in BALF cells before and after one and two weeks of treatment with GC was evaluated by qPCR. Bars represent means ± SEM. * Significant difference from controls at the same time (*p < 0.05, **p < 0.001). **b** Blood neutrophils were incubated 24 h with or without 100 ng/ml reIL-17, cytocentrifuged and stained with a modified Wright–Giemsa solution, and analyzed using the NETs score. **c** Representative immunofluorescence images were taken one hour after PMA treatment, staining with Histone H3 (green), MPO (red) and DAPI (blue). Higher-magnification views show NETs formation, demonstrated by the co-localization of Histone H3 (green), MPO (red) and DNA (blue). Scale bar represents 20 μm. **d** Neutrophils isolated from blood were seeded in the presence of 200 nM of PMA, ± 100 ng/ml reIL-17 and ±10^−6^ M dexamethasone for 1 hour. Cells were stained with 2.5 μM of DNA-staining dye Sytox Orange. Random images taken by a blinded evaluator and NETs quantification were assessed. Bars represent the fold change, expressed as the mean ± SD relative to the PMA-stimulated condition. * Significant differences from PMA (*p* < 0.05)

IL-17 mRNA was overexpressed in the airways of asthmatic horses (Fig. 6a) when compared to controls (*p* = 0.007), and was unaffected by GC.

### IL-17 inhibits the release of NETs induced by PMA

As IL-17 was previously found to be associated with NETs formation [21], we next investigated whether the persistence of IL-17 in the airways of asthmatic horses, despite GC treatment, could influence the production of NETs. In preliminary experiments, we found that IL-17 decreases rather than increases NETs formation by neutrophils (Fig. 6b); we thus developed a live assay to determine whether this effect remains present in activated neutrophils. With a concentration of 200 nM, we found that PMA induce NETs formation (Fig. 6c). As noticed in preliminary experiments, we did observe that IL-17 (*p* = 0.0124) significantly inhibits PMA-induced NETs formation, as well as dexamethasone (*p* = 0.0216) (Fig. 6d).

## Discussion

NETs participate in antimicrobial defense; however, their persistence in tissues can result in host damage. NETosis has been shown to contribute to lung pathology by promoting airway obstruction, alveolar capillary damage and disruption of host proteins and cellular matrix [31, 32]. The recently recognized neutrophilic asthma phenotype is considered a major health concern as affecting >50% of severe human asthmatics [33, 34]. These patients are often poorly responsive to therapy, have fixed airflow obstruction and an accelerated decline in lung function. Severe equine asthma shares numerous similarities with human neutrophilic asthma [35], and was studied here to perform experiments not possible in humans because of ethical considerations. Interestingly, NETs are found to be sensitive to non-steroidal anti-inflammatory drugs in vitro but were reported to be unaffected by GC [19]. As in vitro findings do not always reflect in vivo effects, we evaluated whether NETs formation is insensitive to GC through an IL-17 pathway in an equine model of asthma. However, to the contrary, we observed that GC decreases PMA-induced NETs formation in vitro and also in vivo in the lungs of severe asthmatic horses. This effect was observed despite the persistence of neutrophils within the airways and was independent of the activation state of neutrophils and the persistence of IL-17 in asthmatic airways. Taken together, these results suggest that NETs formation is sensitive to GC and independently regulated by neutrophil recruitment within the airways and their activation.

We first confirmed the presence of NETs in the airways of asthmatic horses [36], as observed in human asthmatic patients [37]. Extracellular DNA quantification was difficult to determine in BALF using usual fluorometric methods or agarose gel electrophoresis since DNA binds to tenacious mucus that is difficult to dissolve without damaging the NETs (data not shown). Considering this, we developed and adapted a score to quantify NETs in airway secretions, estimating the surface of the microscopic field occupied by NETs, with unbiased point counting, as this parameter seems to be an indicator of inflammation [38]. In BAL cytospin, NETS were identified only in the peripheral areas of the sildes. We suggest that neutrophils undergo morphological changes with NETs secretion, that lead to their accumulation in the peripheral areas on the slides with the centrifugal force. As the NETs score was zero for the central areas, we only reported NETs scores for peripheral ones.

Importantly, we observed that dexamethasone inhibits NETs formation in both asthmatic and control horses. To the best of our knowledge, this is the first study investigating the role of GC in NETosis in vivo. In agreement with this finding, and contrary to a previous report [19], dexamethasone inhibited in vitro the PMA-induced NETs formation in the present study. While species differences may have contributed to these contrasting results, our experimentation allowed us to detect only NETs neoformation following a shorter PMA simulation period. After one hour, we observed, chronologically, adherence and flattening, vacuolization, and intracellular chromatin decondensation. Nevertheless, as NETs formation was not attenuated after one week of treatment, this delayed response, combined with the previous in vitro findings [19], suggests an indirect effect of GC on the lung and that the inflammatory microenvironment in the airways may be contributing to the delayed effect of GC.

The migration of neutrophils from the blood to the inflammation site involves a complex regulation of surface adhesion proteins and expressions of activation markers. The tight adhesion and transendothelial migration of granulocytes mediated by β2 integrins, including CD11b (CR3, or Mac1) and aminopeptidase CD13 [39], are required for blood neutrophils to reach the airway lumen. We, therefore, investigated whether NETs inhibition was specific or resulting from an overall downregulation of neutrophil activation by GC. We found that dexamethasone reduced the expression of CD11b in blood neutrophils but not in pulmonary neutrophils of asthmatic horses, and that a similar trend occurred for CD13. As CD11b [40] and CD13 [41] are cell surface markers typically expressed upon neutrophil activation, these results suggest a distinct pro-inflammatory milieu for neutrophils in airways and blood, regardless of the disease status. These findings also support the previous report [42] stating that following the bronchial instillation of endotoxins, dexamethasone has a more potent anti-inflammatory effect in the blood than in the lungs, in healthy human volunteers.

IL-17 mRNA was increased in BAL cells of asthmatic horses and was unaffected by GC. In human studies, GC had conflicting effects on IL-17 production (6, 32) and, therefore, our IL-17 mRNA results within the lung during a GC therapy need to be investigated at the protein level. Furthermore, to our knowledge, while IL-17 has been implicated in steroid-resistant neutrophilic asthma [43–45], its possible contribution to NETs formation in asthma has not been investigated. In this study, we observed that IL-17 decreased NETs formation. These results were unexpected, as IL-17 was shown to be associated with NETs formation in psoriasis lesions [46]. These findings point to a link between IL-17, NETosis and GC insensitivity in severe neutrophilic asthma [46], which is intriguing since we reported that IL-17 activates and increases the survival of neutrophils in vitro, which are unresponsive to GC [24]. Thus, considering that IL-17 decreases NETs formation and that neutrophils also die by NETosis*,* it remains possible that the IL-17 pathway resistant under GC contributes to the persistence of neutrophils in the asthmatic airways. Also, although IL-17 decreases NETs formation in vitro, it also promotes the production of IL-8 within the lung tissue [24]. As IL-8 induces NETs formation [47], the balance of IL-8 inducing NETs release may be opposed by the IL-17 inhibitory response. However, the effects IL-17 have on NETs formation in vivo likely depend on the stimuli applied and therefore, the response within the lung microenvironment may differ from the inhibition we observed in vitro.

Our results also demonstrate that GC decrease the apoptosis of airway neutrophils. Studies have reported that while in vitro*,* neutrophil apoptosis is delayed by GC [3, 48, 49], no studies have compared the level of apoptosis in vivo before and following GC treatment in asthmatic and in healthy subjects. As dexamethasone did not affect the activation markers of pulmonary neutrophils, but decreased neutrophil apoptosis in both healthy and asthmatic animals, neutrophils’ response to GC appear to be specific and not influenced by asthmatic inflammation. However, apoptosis levels are relatively low (around 5%) and probably contribute minimally to the airway neutrophilia persistence in asthmatic animals. Further investigations using larger sample sizes need to be performed to confirm these results.

## Conclusions

In conclusion, the results of this study indicate that GC have a different effect on blood and airway neutrophils. A two-week treatment with GC decreased NETs formation without decreasing activation markers CD11b and CD13 in pulmonary neutrophils. Further studies are needed to determine whether this overall effect is favorable or harmful to the resolution of asthmatic inflammation. Our results suggest that the Th17 pathway may play a role in decreasing NETosis; however, additional studies are required to determine its overall contribution to asthma.

## Additional files

Additional file 1: Figure S1. NETs score validation in BALF cytology. A) The scores were first validated by comparing the staining of the same extracellular DNA regions using Sytox Orange as positive control and Wright-Giemsa. Scale bars represent 100 μm. (B) Frame illustrating the software used for unbiased point counting analysis of the BALF cytology. Bottom: a magnification of the yellow area in the small up panel. Blue crosses were used as probes for NETs volume; blue crosses with green circles were used as probes for reference volume. An unbiased point counting technique using grids with 2304 crosses (randomly selected) per field was performed. Crosses marked with the letter A for NETs/MPO were used for counts. Mate method NETs volume density (Vv _NET/MPO_) was calculated for 7 horses as follow: Vv_NET/MPO_ = ΣP_NET/MPO_/P_ref_ *Total Vv, where ΣP_NET/MPO_ represents the sum of the points crossing onto a NET where MPO wrap around it, Pref indicated the total crosses number per field (2304) and Total Vv the known and fix field area (0,3795mm^2^). A minimum of 200 points was counted for ASM from at least two biopsies per horse. (C) Correlation between our developed score and quantitative unbiased method (*r* = 0.79; *p* = 0.05). (PDF 5456 kb)

Additional file 2: NETs score validation. (DOCX 120 kb)

## Abbreviations

ASM: Airway smooth muscle
BALF: Bronchoalveolar lavage fluid
CD11b: Cluster of differentiation 11b
CD13: Cluster of differentiation 13
CD18: Cluster of differentiation 18
COPD: Chronic obstructive pulmonary disease
CS: Glucocorticosteroids
FACS: Flow cytometry
FBS: Fetal bovine serum
ICS: Inhaled glucocorticosteroids
NETs: Neutrophil extracellular traps
PBS: Phosphate-buffered saline
PMN: Polymorphonuclear neutrophil
