# Supplementary figures and images for: Neutrophil extracellular traps are downregulated by glucocorticosteroids in lungs in an equine model of asthma
Source: Respir Res. 2017 Dec 12;18:207. doi: 10.1186/s12931-017-0689-4 (PMC5727947; doi:10.1186/s12931-017-0689-4)

A

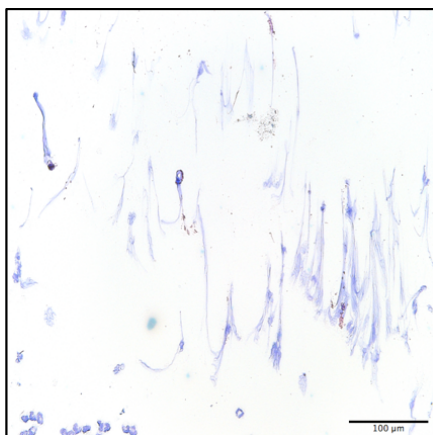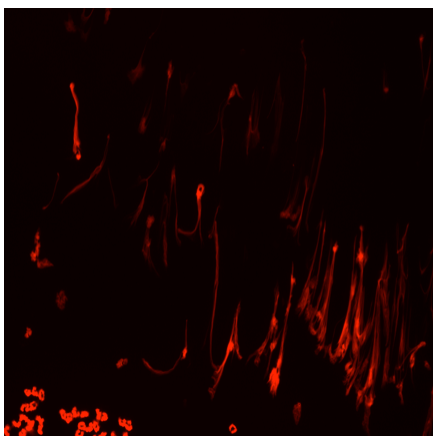

B

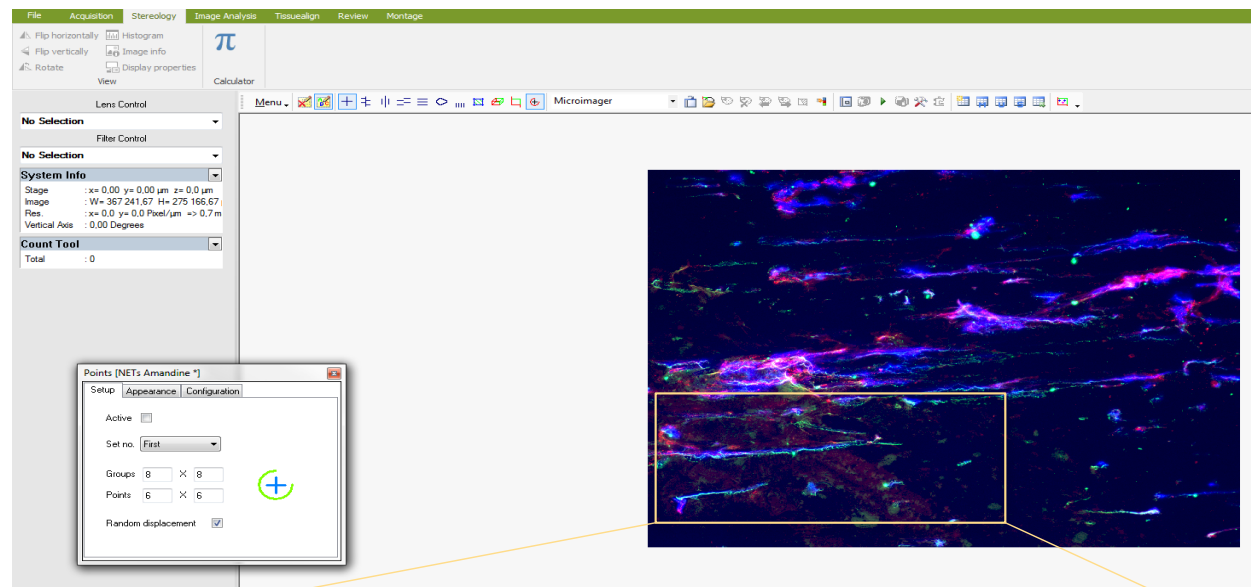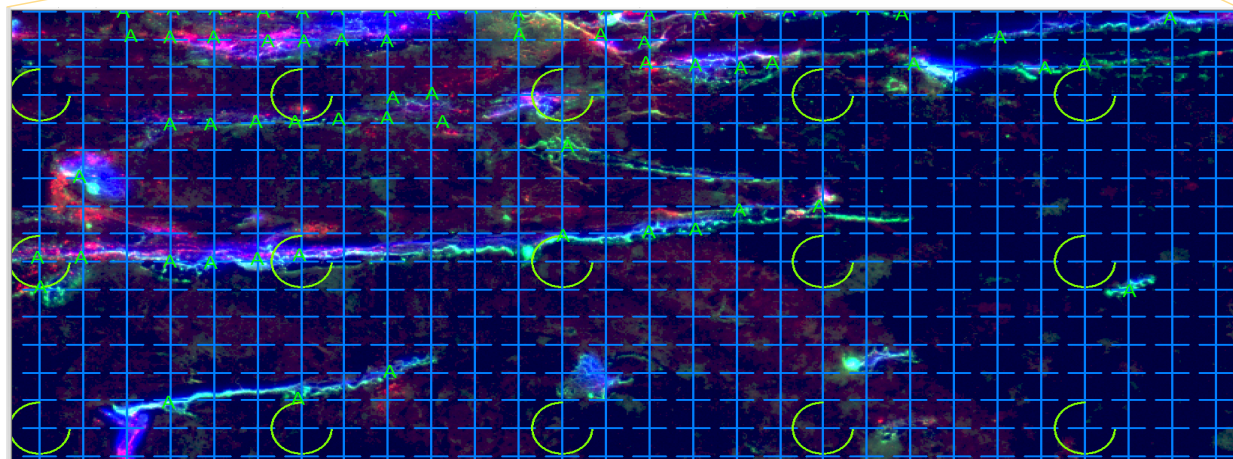

C

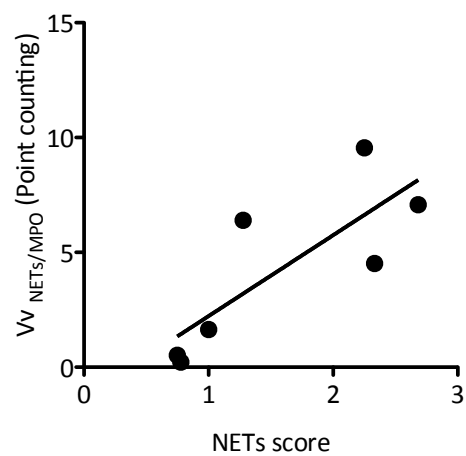

Figure 1

Supplement: Supplementary file 1 — NETs score validation in BALF cytology. A) The scores were first validated by comparing the staining of the same extracellular DNA regions using Sytox Orange as positive control and Wright-Giemsa. Scale bars represent 100 μm. (B) Frame illustrating the software used for unbiased point counting analysis of the BALF cytology. Bottom: a magnification of the yellow area in the small up panel. Blue crosses were used as probes for NETs volume; blue crosses with green circles were used as probes for reference volume. An unbiased point counting technique using grids with 2304 crosses (randomly selected) per field was performed. Crosses marked with the letter A for NETs/MPO were used for counts. Mate method NETs volume density (Vv NET/MPO) was calculated for 7 horses as follow: VvNET/MPO = ΣPNET/MPO/Pref *Total Vv, where ΣPNET/MPO represents the sum of the points crossing onto a NET where MPO wrap around it, Pref indicated the total crosses number per field (2304) and Total Vv the known and fix field area (0,3795mm2). A minimum of 200 points was counted for ASM from at least two biopsies per horse. (C) Correlation between our developed score and quantitative unbiased method (r = 0.79; p = 0.05). (PDF 5456 kb) [file 12931_2017_689_MOESM1_ESM.pdf]
