# Supplementary material for: Neutrophil extracellular traps are downregulated by glucocorticosteroids in lungs in an equine model of asthma
Source: Respir Res. 2017 Dec 12;18:207. doi: 10.1186/s12931-017-0689-4 (PMC5727947; doi:10.1186/s12931-017-0689-4)
Supplement: Supplementary file 2 — NETs score validation. (DOCX 120 kb) [file 12931_2017_689_MOESM2_ESM.docx]

Additional file- NETs score validation

**Score validation** - To confirm the NET detection on BALF cytospin, the score was first corroborated by comparing the staining of the decondensed chromatin filaments (Supplementary Figure 1A) using Wright-Giemsa and Sytox Orange. A standard immunofluorescence assay was also used in which [1-3] DNA, histone H3 and MPO [3] were visualized simultaneously by immunofluorescence and analyzed using an unbiased point counting approach (Supplementary Figure 1B). Volume density (Vv _NET/MPO_) was calculated for seven horses and results were correlated with the developed score (Supplementary Figure 1C). Using a 20X objective, the percentage of neutrophil-releasing NETs was quantified blind by evaluating neutrophils displaying expanded nuclei (nuclei without lobulation and exceeding the normal average diameter of DNA staining (10 μm)) and releasing DNA fibers colocalized with MPO in at least five random microscope fields. The image files were analyzed with the Visopharm's newCAST™ version 6·0 software (Visiopharm, Horsholm, DNK). NETs volume density (Vv _NET/MPO_) was calculated for 7 horses as follows:

**Vv_NET/MPO_=ΣP_NET/MPO_ / P_ref_ *Total Vv**

Where ΣP_NET/MPO_ represents the sum of the points crossing onto a NET where MPO wrap around it, P_ref_ indicated the total cross numbers per field (2304) and Total Vv the known and fix field area (0,3795mm^2^). A minimum of 200 points was counted for each field.

**REFERENCES**

1. Brinkmann, V., et al., **Automatic quantification of in vitro NET formation.** *Front Immunol*, 2012. **3**: p. 413.

2. Kessenbrock, K., et al., **Netting neutrophils in autoimmune small-vessel vasculitis.** *Nat Med,* 2009. **15**(6): p. 623-5.

3. Papayannopoulos, V., et al., **Neutrophil elastase and myeloperoxidase regulate the formation of neutrophil extracellular traps***.* *J Cell Biol*, 2010. **191**(3): p. 677-91.
